# Microchemical analysis of Leonardo da Vinci’s lead white paints reveals knowledge and control over pigment scattering properties

**DOI:** 10.1038/s41598-020-78623-5

**Published:** 2020-12-10

**Authors:** Victor Gonzalez, Selwin Hageraats, Gilles Wallez, Myriam Eveno, Elisabeth Ravaud, Matthieu Réfrégiers, Mathieu Thoury, Michel Menu, Didier Gourier

**Affiliations:** 1 CNRS, Institut de Recherche de Chimie Paris (IRCP), Chimie ParisTech, PSL University, 75005 Paris, France; 2 Science Department, Rijksmuseum, Hobbemastraat 22, 1071 ZC Amsterdam, The Netherlands; 3 Centre de Recherche et de Restauration des Musées de France (C2RMF), Palais du Louvre, 75001 Paris, France; 4 IPANEMA, CNRS, Ministère de La Culture, Université de Versailles Saint-Quentin-en-Yvelines, Université Paris-Saclay, BP48 St. Aubin, 91192 Gif-sur-Yvette, France; 5 Synchrotron SOLEIL, L’Orme Des Merisiers, BP48 St. Aubin, 91192 Gif-sur-Yvette, France; 6 Sorbonne Université, UFR926, 75005 Paris, France

**Keywords:** Analytical chemistry, Characterization and analytical techniques

## Abstract

Leonardo da Vinci (1452–1519) is a key artistic and scientific figure of the Renaissance. He is renowned for his science of art, taking advantage of his acute observations of nature to achieve striking pictorial results. This study describes the analysis of an exceptional sample from one of Leonardo’s final masterpieces: *The Virgin and Child with St. Anne* (Musée du Louvre, Paris, France). The sample was analyzed at the microscale by synchrotron-based hyperspectral photoluminescence imaging and high-angular X-ray diffraction. The results demonstrate Leonardo’s use of two subtypes of lead white pigment, thus revealing how he must have possessed a precise knowledge of his materials; carefully selecting them according to the aesthetical results he aimed at achieving in each painting. This work provides insights on how Leonardo obtained these grades of pigment and proposes new clues regarding the optical and/or working properties he may have tried to achieve.

## Introduction

Leonardo da Vinci (1452–1519) is a key figure of the Renaissance^1,2^. His constant search for novelty allowed him to develop new painting techniques he was the only one to master, such as the famous sfumato^3^. However, while the exceptional pictorial results obtained by Leonardo can still be admired today, he left no precise indications on the recipes he used to achieve them. 500 years after the completion of his artworks, only chemical analysis can reveal the materials and secrets of the Master^4–6^.

**Figure 1:**
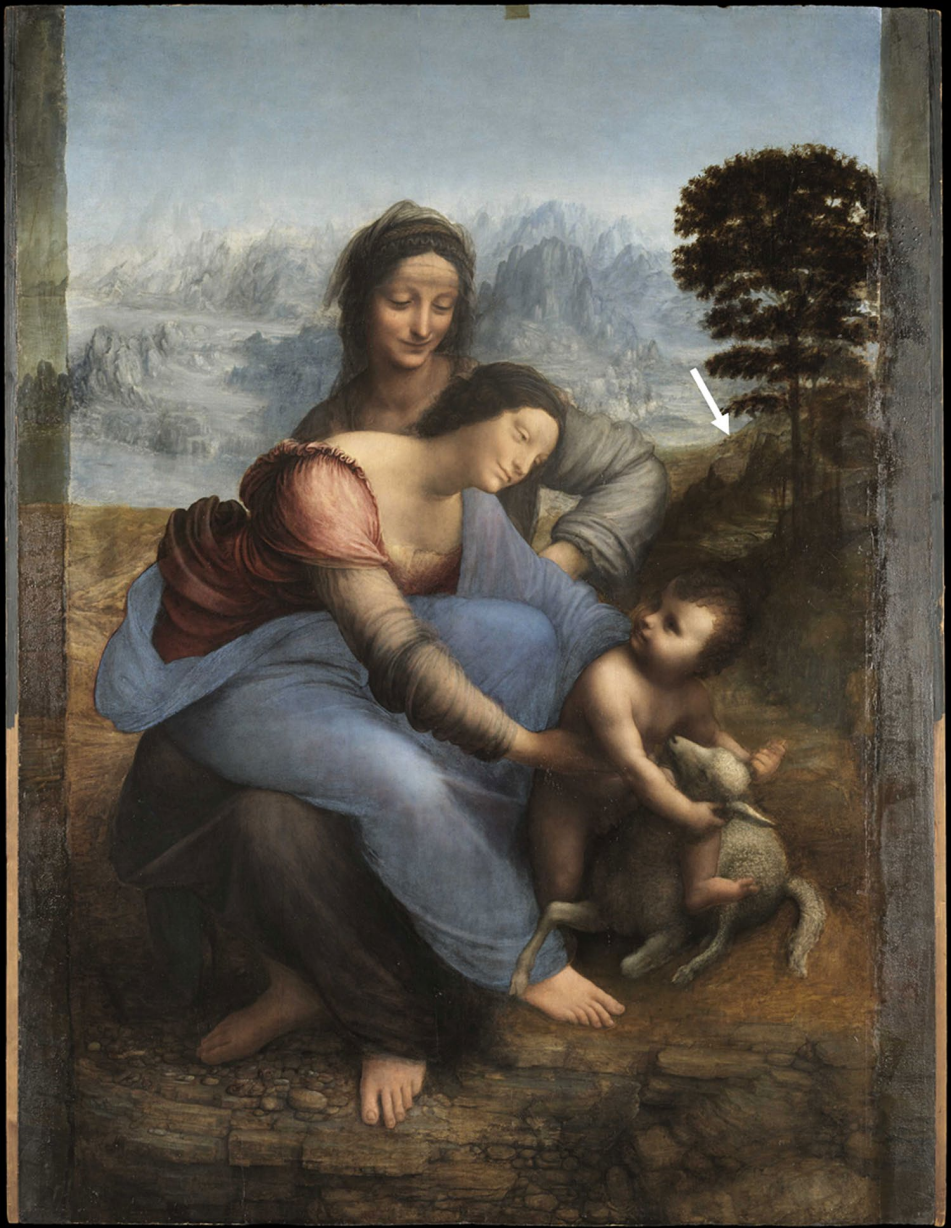
Leonardo da Vinci, *The Virgin and Child with St. Anne*, (168 × 130 cm), 1503–1519, Musée du Louvre. Image courtesy of C2RMF. The white arrow indicates the location of sampling.

Painted between 1503 and Leonardo’s death in 1519, the *St. Anne* (Fig. 1) is considered one of his ultimate masterpieces along with the *Mona Lisa*. The painting is in the collection of the Musée du Louvre and was studied in the framework of its restoration at the *Centre de Recherche et de Restauration des Musées de France* (C2RMF) in 2007^7^. In this context, several microsamples were collected to help in deciphering the materials and techniques of Leonardo. Among those samples, one was taken from the composition background, near the frontier between the brown rocky landscape and the mountains appearing in a blue haze (white arrow in Fig. 1).

**Figure 2:**
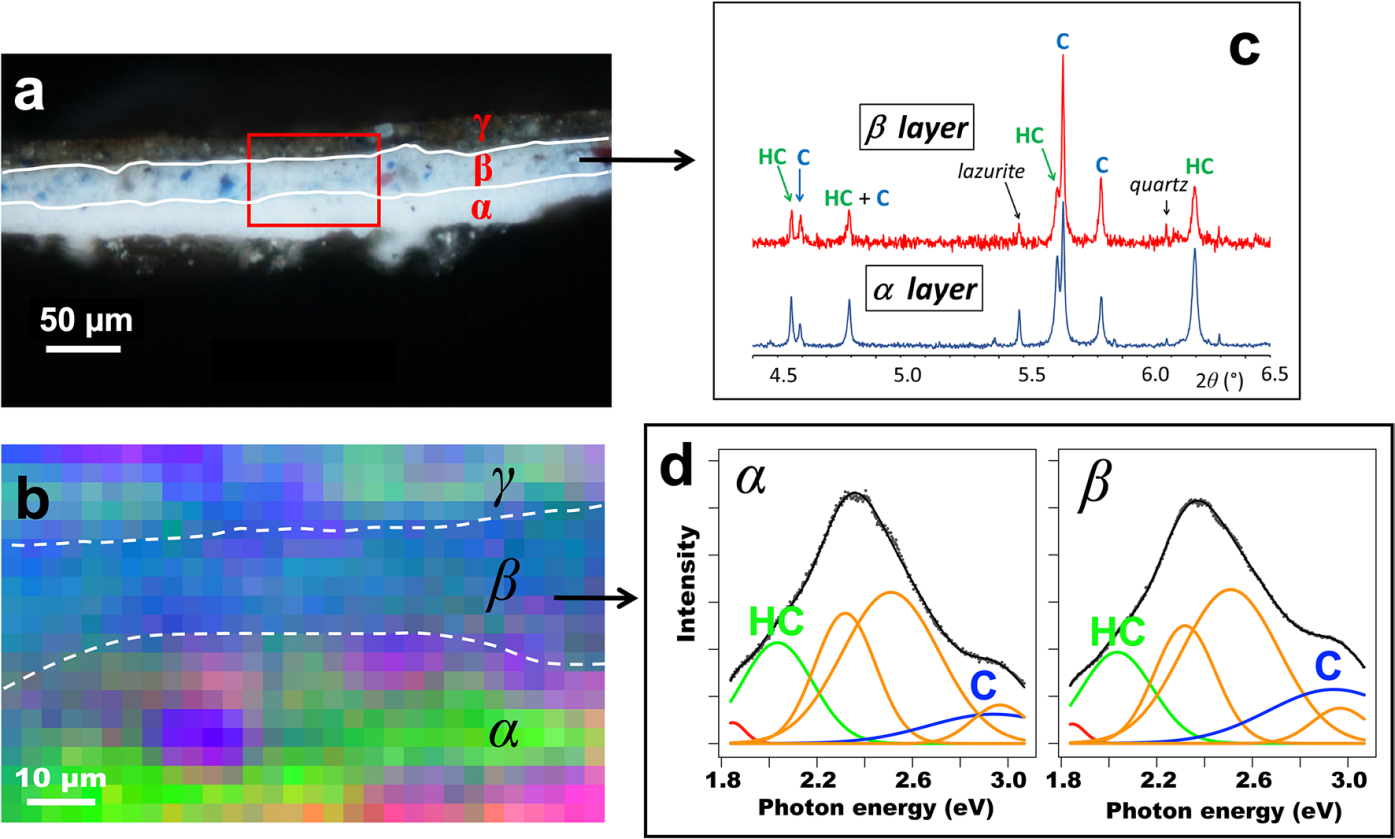
(**a**) Dark-field microscopy image of the *St. Anne* cross-section showing three layers denoted α, β and γ. The red rectangle indicates the region analyzed by SR-µ-PL. (**b**) Unfolded false-color representation of the SR-µ-PL map. The amplitude matrices of the Gaussian contributions at 1.85, 2.03, 2.94 eV are shown as the red, green, and blue channel respectively. Dashed lines indicate the interfaces between layers α, β and γ. (**c**) Zoom of a portion of the XRD patterns for layers α (blue line) and β (red line). Bragg positions for C and HC are indicated. (**d**) Average spectral fit of SR-µ-PL for layers α and β. Red, blue and green components are emission bands that were mapped out in (**b**).

The optical image of the stratigraphy of this sample (Fig. 2a) shows that the blue hue of the paint was achieved using a very small amount of coloring material: only a few particles of ultramarine (a blue pigment composed of the mineral lazurite) were dispersed in an almost entirely white layer (layer β). SEM–EDX allowed to detect also a few grains of silicates and red lake pigments. This layer was painted on top of the *imprimitura* (layer α) solely composed of white material in oil. A ground layer of gesso is present under the *imprimitura*. Finally, a layer with a more complex composition—notably containing earth and copper pigments, quartz, lead–tin yellow and alumino-silicates—was painted on top (layer γ). The white material present in both layers was identified as lead white, one of the most widely used pigments in easel paintings prior to the twentieth century.

Lead white is composed of a mixture of two crystalline phases^8^, namely cerussite (C) PbCO_3_, and hydrocerussite (HC) Pb_3_(CO_3_)_2_(OH)_2_. In aqueous media, the formation of the two phases is pH-dependent: C being stable in an acidic (pH < 6), and HC in an alkaline (8 < pH < 10) environment^9^. The synthesis of lead white during the Renaissance was based on the dry corrosion of metallic lead by vinegar vapors (containing acetic acid) in the presence of CO_2_, O_2_ and H_2_O^10^. Lead carbonates (C and HC) were formed at the surface of lead foils, then collected and sold to painters. As the same synthesis process (often referred to as stack process) was used since antiquity, one might expect to find a nearly identical material throughout works of art. However, historical sources reveal that the obtained raw material could be modified by the paint manufacturers or the painters themselves. Numerous post-synthesis treatments were thus used. According to the degree of sophistication of the fabrication process of the pigment, different qualities were obtained and sold at various prices to the painters before being mixed with the organic binder and employed in a painting^11,12^. Unfortunately, Leonardo, as his contemporaries, never revealed what materials he used in his artworks. The sample of the *St. Anne* with its two adjacent β and α lead white layers thus appears as a unique opportunity to provide new clues into Leonardo’s technique, and the possible perceptive effects he wanted to achieve.

Today, quantifying the ratio of the two constitutive phases of lead white appears to be the most efficient way to provide clues on the pictorial choices of the Old Masters^13^. Until recently, the only method used to achieve this was X-ray diffraction (XRD). In particular, synchrotron-generated X-ray micro-beams allow to quantify those compounds inside micrometric cross-sections^14^. However, radiation damage due to long acquisition times and the need to use synchrotron facilities pose serious challenges for analysis on precious historical samples. In a recent study, it was shown that HC and C can also be discriminated on the basis of their photoluminescence (PL) properties upon excitation in the deep-UV (5 eV, 250 nm)^15^. Still, due to complex emission behaviors, the ability to discriminate lead white phases based on PL has so far not been demonstrated in a micro-imaging application. This work demonstrates how semi-quantitative information can be obtained on the lead white composition in the different paint layers of the *Ste. Anne* microsample by employing deep-UV excited hyperspectral photoluminescence microspectroscopy and a dedicated spectral unfolding procedure. Beyond the investigation of Leonardo’s painting technique, an important goal of this work was to establish photoluminescence—through validation with synchrotron-XRD—as a viable non-invasive tool for the spatially resolved characterization of lead white in historical microsamples.

## Results

### Synchrotron high-angle resolution X-ray diffraction (SR-HR-XRD)

Two fragments about 50–80 µm in size were collected from layers α and β of the *St. Anne* sample for XRD analysis at the ID22 beam-line (ESRF, Grenoble, France). One fragment is made of pure layer β, while the other is made primarily of the *imprimitura* layer α. It must be noted that this second sample also contains a minor part of layer β, as was concluded from the detection of small amounts of lazurite. These two fragments will be hereafter referred to as the β fragment and α fragment, respectively.

Zooms of the diffractograms collected on the α and β fragments in the angular range 4.5 ≤ 2θ (°) ≤ 6.5 are shown in Fig. 2c. Here, due to the low amount of lead white pigment in layer β, the diffraction pattern of the β fragment exhibits a lower signal-to-noise ratio than the one of the α fragment.

The full XRD patterns with corresponding Rietveld refinements are given in Supplementary Information. The quantitative phase analysis of XRD diagrams (Fig. 2c) shows the prevalence of cerussite in the β fragment (R = HC/(HC + C) = 46 ± 2 w%) and of hydrocerussite in the α fragment (R = HC/(HC + C) = 65 ± 2 w%). However, taking into account that the α fragment is contaminated by some β component, the actual HC content of the former is likely to be somewhat higher than the experimental value. For the sake of realism, the standard uncertainties have been multiplied by Bérar’s factor^16^ computed by FullProf.

In addition to layer compositions, HR-XRD can provide information about the average morphologies of lead white crystallites. The broadening of a diffraction peak β = FWHM − FWHM_*ins*_, where FWHM_*ins*_ is the instrumental width, can be considered as the sum of size and strain-induced contributions *β*_*L*_ and *β*_*S*_. Assuming *β*_*L*_ = 0.9*λ*/Lcos*θ* (“Scherrer broadening” formula) and *β*_*S*_ = 4*ε* tan*θ* (from the logarithmic differentiation of Bragg’s law), where λ is the wavelength, *θ* the Bragg’s angle, *L* the mean crystallites size perpendicular to the corresponding (*hkl*) planes, and *ε* the strain coefficient, the broadening of diffraction peaks are given by the Williamson & Hall’s (WH) linearized form^17^:

1 $$ \beta\;{\text{cos}}\;\theta \, = \, 0.{9}\lambda \, /L + { 4}\varepsilon {\text{sin}}\theta $$

**Figure 3:**
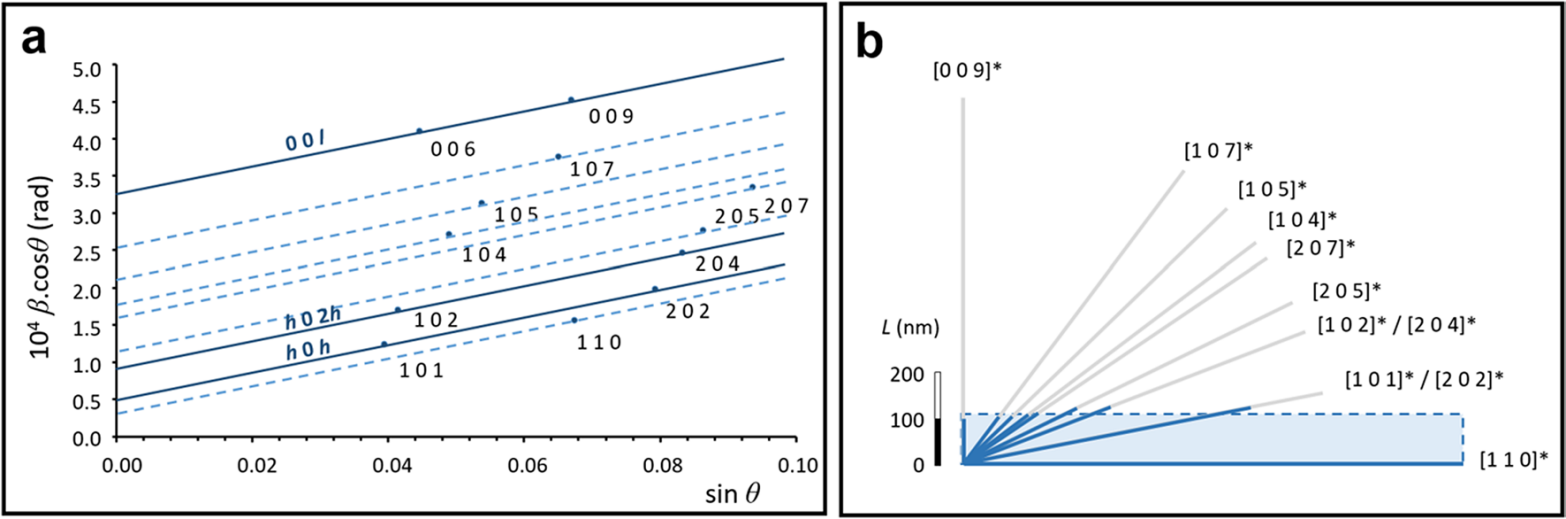
(**a**) Williamson-Hall (WH) analysis for HC of the α fragment. The *h* 0 h, *h* 0 2* h* and 0 0 l lines exhibit a similar slope that was applied to the other reflections for the determination of the size broadening. (**b**) (a*, c*) cross section (shaded area) of a typical HC platelet of the α fragment, based on crystal dimensions computed by Williamson–Hall analysis.

By plotting *β* cosθ = f (sinθ), the crystallites size *L* and the strain coefficient ε can be inferred from the y-intercept and from the slope, respectively. In the case of anisotropic crystals like HC, straight lines can only be plotted from reflections of the same plane at different orders *n* (*nh nk nl* series). The crystal geometry can then be rebuilt by reporting the corresponding *L* lengths along the planes’ perpendicular directions. The WH analysis of the HC phase in the α layer (Fig. 3a) allows to determine the morphology of the average crystallite as a platelet 110 ± 20 nm thick, with a 10:1 aspect ratio (Fig. 3b) and a low and quasi-isotropic strain coefficient *ε* = 4.7 ± 0.2 × 10^–4^ computed from reflections of different orders. Implementing this procedure proves more delicate for HC in layer β because of the low lead white content of this layer, but the peak widths are not significantly different. Concerning C, the dispersion of the experimental points is not apparently ruled by the orthorhombic symmetry, but maybe by the twinning commonly observed in this structure that results in a slight Lorentzian broadening. Fitting the whole diagram with a single straight line yields a near-zero Scherrer broadening, corresponding to C particles larger than 500 nm (see Supplementary Information).

### Synchrotron deep UV-excited photoluminescence (SR-µ-PL)

Having measured differences in the HC content of the α and β fragments by SR-HR-XRD, the possibilities of SR-µ-PL spectroscopy and imaging to determine lead white compositions throughout the whole *St. Anne* cross-section was explored. Figure 2d shows an average spectral fit of the PL spectrum in layer β, excited at 5 eV. This photon energy corresponds to the ^3^P_1_(6s6p) ← ^1^S_0_(6s^2^) transition of Pb^2+^ ions^15,18^. The superposition of six transitions with Gaussian profiles (centered at 1.85 eV, 2.03 eV, 2.32 eV, 2.51 eV, 2.94, and 2.97 eV) was necessary to give a satisfactory fit. It was previously shown that the excitation of lead white at 5 eV produces emission with very large Stokes shifts, centered at 2.8–2.9 eV for C and at 2.0–2.1 for HC^15^. Both emissions were attributed to ligand-to-metal (CO_3_-to-Pb) charge transfer transitions^15,19^. Thus, we attribute the PL bands at 2.03 eV (green curve on Fig. 2d) and 2.94 eV (blue curve on Fig. 2d) to emission of HC and C, respectively. The weak red emission at 1.85 eV is at the lower limit of the experimental spectrum and is typical of the ^4^T_1_ → ^6^A_1_ emission of Mn^2+^ impurities, which occurs around 1.9 eV in cerussite^20^. Considering the images shown in supporting information (Fig. S3) the emission of the PL band at 2.32 eV was found to spatially correlate to that of the band at 2.03 eV. This means that the emission band at 2.32 can likely also be attributed to HC, together forming the broad emission feature centered around 2.15 eV that was previously found by Gonzalez et al. on pure hydrocerussite samples^15^.

Finally, without any other plausible origin, the bands at 2.51 and 2.97 eV (two rightmost orange curves in Fig. 2d) were attributed to the oxidized drying oil binding medium. The two main arguments are (1) the knowledge that drying oils do exhibit photoluminescence in the visible^21,22^ and (2) the observation that the emission intensity of these two bands is distributed more or less homogenously throughout the three paint layers (see supporting information, Fig. S3).

The amplitudes of the Gaussian bands at 1.85, 2.03 and 2.94 eV were mapped out, intensity stretched, and combined into a false-color RGB image, displayed in Fig. 2b. Chemical contrast between the different layers can clearly be visualized: the bottom layer α exhibits a green signature dominated by the 2.03 eV emission of HC, whereas the middle layer β exhibits a blue signature dominated by the emission band of C at 2.94 eV (Fig. 2b).

The HC and C content per layer could not be quantified directly by PL, due to a lack of information about the quantum yields and molecular absorption coefficient of cerussite and hydrocerussite. However, a metric Γ is proposed that still allows to relate the unfolded photoluminescence emission amplitudes to the HC content R = HC/(HC + C). The emission amplitude *A*_C_ at 2.94 eV for cerussite (and similarly *A*_HC_ at 2.03 eV for hydrocerussite) is proportional to the quantum yield Φ_C_, the molecular absorption coefficient ε_C_ at the excitation photon energy 5 eV, and the concentration [C], i.e. $A_{C} \propto \Phi_{C} \,\varepsilon_{C} \,\left[ C \right]$. Under the assumption that the Φ and ε parameters are the same in layers α and β for a given lead white component (HC or C), the ratio between emission amplitudes in the two different paint layers does not depend anymore on Φ and ε, and is equal to the ratio between the concentrations of C in the two layers:

2 $$ \frac{{\left( {A_{C} } \right)_{\beta } }}{{\left( {A_{C} } \right)_{\alpha } }} = \frac{{\left[ C \right]_{\beta } }}{{\left[ C \right]_{\alpha } }} $$

The same equation holds for hydrocerussite, where the subscript C is replaced by HC. Based on this relation, the metric Γ is then defined as follows:

3 $$ \Gamma \equiv \frac{{\left( {{{\left[ {HC} \right]} \mathord{\left/ {\vphantom {{\left[ {HC} \right]} {\left[ C \right]}}} \right. \kern-\nulldelimiterspace} {\left[ C \right]}}} \right)_{\alpha } }}{{\left( {{{\left[ {HC} \right]} \mathord{\left/ {\vphantom {{\left[ {HC} \right]} {\left[ C \right]}}} \right. \kern-\nulldelimiterspace} {\left[ C \right]}}} \right)_{\beta } }} = \left( {\frac{{A_{HC} }}{{A_{C} }}} \right)_{\alpha } \left( {\frac{{A_{C} }}{{A_{HC} }}} \right)_{\beta } $$

The value Γ ≈ 2.1 is obtained from the average photoluminescence emission amplitudes of layers α and β (see supporting information). Γ is also related to the HC content R measured by HR-XRD by:

4 $$ \Gamma \equiv \frac{{\left( {{{\left[ {HC} \right]} \mathord{\left/ {\vphantom {{\left[ {HC} \right]} {\left[ C \right]}}} \right. \kern-\nulldelimiterspace} {\left[ C \right]}}} \right)_{\alpha } }}{{\left( {{{\left[ {HC} \right]} \mathord{\left/ {\vphantom {{\left[ {HC} \right]} {\left[ C \right]}}} \right. \kern-\nulldelimiterspace} {\left[ C \right]}}} \right)_{\beta } }} = \frac{{\left( {R/(1 - R)} \right)_{\alpha } }}{{\left( {R/(1 - R)} \right)_{\beta } }} $$

The values Γ ≈ 2.1 derived from hyperspectral SR-µ-PL (Eq. 3) and Γ ≈ 2.2 derived from HR-XRD (Eq. 4) are sufficiently similar to validate our interpretation of PL data and to confirm that layer α is dominated by HC while layer β is dominated by C. The ~ 5% difference in Γ between XRD and PL may be due to a number of factors, including deviations in the assumption that ɸ and ɛ are the same in layers α and β, and an imprecise spatial definition of those two layers in the hyperspectral PL maps. A table recapitulating the ratios obtained with both techniques, SR-HR-XRD and SR-µ-PL for layers α and β is available in supporting information (Table S1).

## Discussion

The combined HR-XRD and SR-µ-PL analysis revealed that the *St. Anne* sample contains two layers with different lead white compositions. This observation raises questions about what the intention of the artist could have been for using two varieties of lead white. Previous analyses of HC and C compositions in a large corpus of Italian Renaissance paintings from the Louvre Museum revealed that in most cases, lead white pigments of this period made by the historical stack process exhibited R = HC/(HC + C) values in the range 60 ≤ R ≤ 80 (w%) and large average particle sizes^14^. This was corroborated by a reconstruction of the synthesis process used in the past, yielding the same composition range 60–80 (w%)^10^ Extending the composition range to R < 50 (w%) required a very long corrosion time and an increased CO_2_ supply, which is limited in the stack process. R > 80 (w%) could be obtained by collecting the pigment before complete consumption of lead, as HC appears and grows before C during the corrosion process^10^. To obtain pigments deviating from the most usual composition range 60 ≤ R ≤ 80 (w%) and with small particle size, the raw material had to be post-processed by the pigment manufacturer or in the painter’s workshop, which must therefore be the result of a conscious choice.

**Figure 4:**
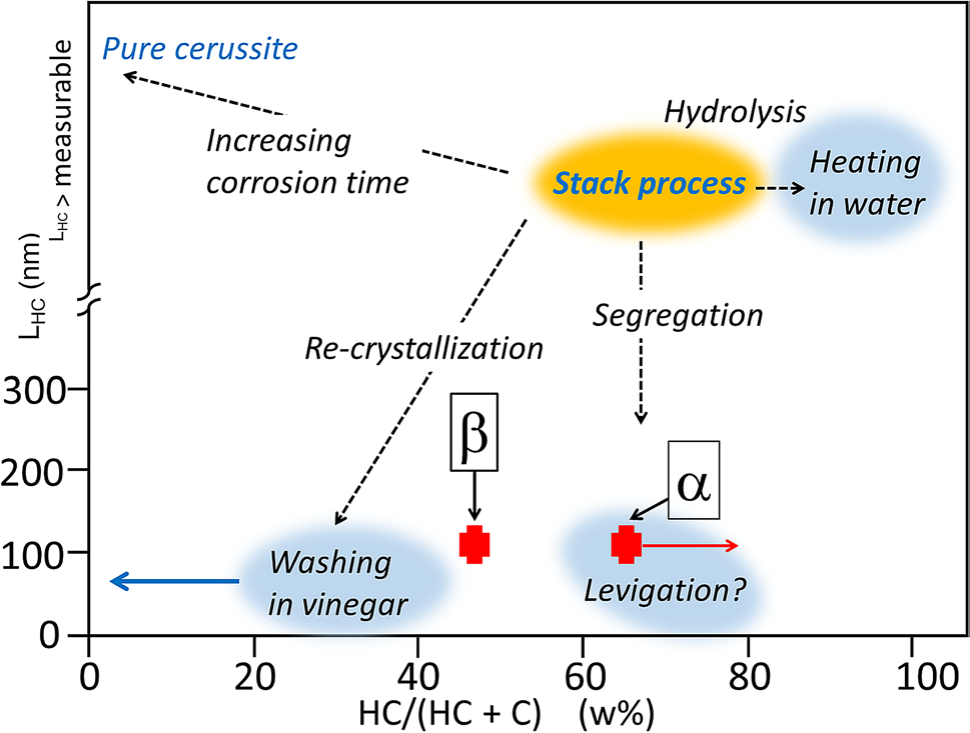
Diagram showing the thickness L_HC_ of HC platelets as a function of lead white composition HC/(HC + C). The colored areas with diffuse contours represent the locations of experimental values measured for 24 samples taken from Renaissance paintings (14). Broad red crosses represent the values measured for fragments α and β in the *St. Anne* sample. The red arrow indicates the fact that the HC content of the α fragment is likely to be slightly underestimated.

A diagram showing the thickness L_HC_ of HC crystallites as a function of the lead white composition is shown in Fig. 4. The data previously collected from 24 samples from Renaissance paintings^14^ are represented by the colored areas in the diagram: in yellow, the primary stack process; in blue, the various secondary post-process treatments. Experimental points for layers α and β of the *St. Anne* sample are shown in red, both falling in a region indicating some form of post-synthesis treatment to obtain crystallites of smaller dimensions.

Ancient recipes indicate two possible ways of obtaining a pigment with submicrometric crystallites: washing of the pigment in vinegar or levigation in water. Regarding the first post-synthesis method—consisting in washing or grinding the lead white in vinegar—historical sources highlight it was a well-attested process during the Renaissance that was said to render the pigment ‘whiter’^23,24^. This increased whiteness is most likely due to the dissolution of the remaining non-corroded dark metallic lead particles in an acidic environment. This acidic treatment also induces the partial dissolution of HC crystallites (and the decrease of their size) and the subsequent precipitation of C, which is more stable in this pH domain. The size of the reprecipitated C particles is also small due to the fast crystallization process^10^. This results in a lead white with R < 50 (w%) and an average particle size in the submicrometer range that is highly polydisperse. For that reason, we were unable to estimate the size of the finest fraction by XRD. The second post-synthesis method consisted in segregating the particles according to their size by levigation in water, without chemical reaction (Fig. 4). This method is not expected to significantly alter the composition of the obtained lead white batch. From Fig. 4 we can therefore conclude that the lead white pigment in layer β was certainly submitted to an acidic treatment.

**Figure 5:**
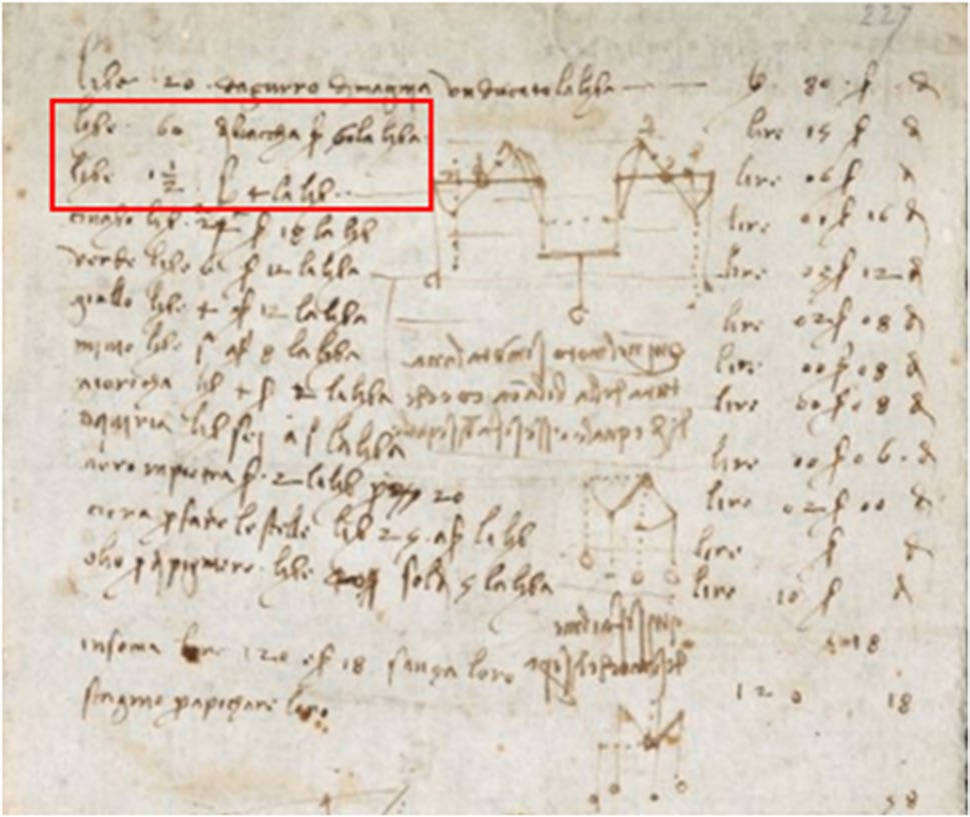
Leonardo da Vinci, Arundel Manuscript 263, detail of f.227v© British Library Board, all rights reserved. The red rectangle indicates two lines where Leonardo mentioned an order for 5 lb of a lead white at 3 *lira* per pound, and of 1.5 lb of another batch of lead white at 4 *lira* per pound.

The question now becomes whether or not Leonardo da Vinci could have had access to different lead white pigments and used them according their specific qualities and properties. Did he buy the lead whites and then treat them himself, or did he buy the two varieties directly from a pigment provider? It is difficult to answer this question. However, the idea of the use by Leonardo of a lead white that was treated post-synthesis to produce a whiter or “higher” grade of pigment is backed up by notes found in the Arundel 263 notebook by Da Vinci, on the recto of folio 228, British Library (Fig. 5)^4^ Here, he lists orders for two different lead whites—one of which is 30% more expensive. This order by the Master for two lead white pigments at two different prices constitutes to our knowledge the earliest mention of distinct lead white qualities in Italian Renaissance history, and gives a proof that Leonardo was using different lead white grades. If this hypothesis is correct, one can imagine that untreated—or “lower” grades pigments without post-synthesis treatment (R > 60w%)—would typically be found in *imprimitura* layers, whereas the treated, or “higher” grades (R < 50 w%) pigments would be found in the upper layers of the painting that are visible to those observing the finished painting. This is in line with the observed HC contents in layers α (*imprimitura*) and β.

**Figure 6:**
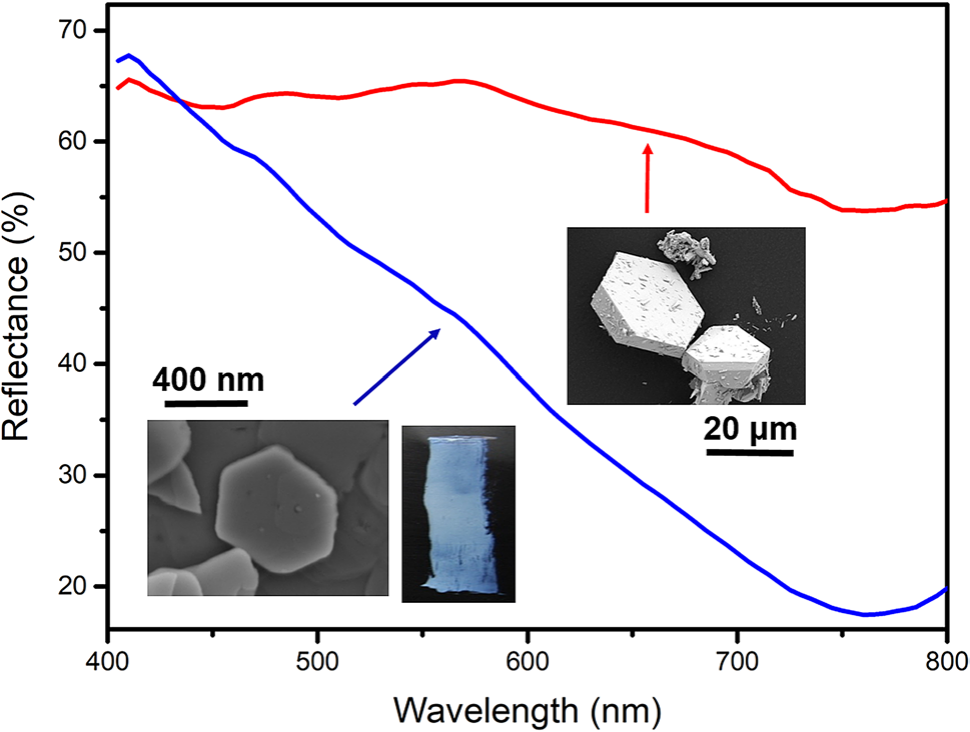
Reflectance spectra of two lead white layers (thickness of 60 µm) com-posed respectively of sub-micrometric (< 400 nm) and large (> 20 µm) HC crystallites. The sub-micrometric pigment exhibits a pronounced decrease of reflectance toward the red region of the visible range, whereas the pigment composed of large crystallites exhibits a nearly constant and high reflectance on the entire visible range. The insets represent the images of HC grains, and the blue hue of the film composed of the finer grade.

We formulate here two hypotheses to explain the choice by Leonardo to use a lead white with very fine particles: The first hypothesis is related to the specific rheological properties Leonardo must have looked for. Particle size is indeed a key parameter regarding the rheological properties of lead white paints, as exemplified in several works^23,25^. Leonardo is very renowned for his use of extremely thin and subtle glaze paint layers. In order to achieve a thin glaze layer, a painter must use a very fine pigment granulometry: this was notably stressed by Leonardo himself^4^ for the ochre pigment. MA-XRF elemental maps on the entire corpus of da Vinci artworks from the Louvre recently highlighted how ochre and lead white pigments were the materials of choice used by the Master to achieve the progressive tone gradients in his flesh tones^4^. We can thus safely assume that he was looking for pigments with very small particle sizes to achieve those effects in his pictorial layers, in this case the β layer of the *Ste Anne*. The second hypothesis is related to the scattering properties of lead white and their relation to the size of the crystallites. Considering the micrometric and submicrometric dimensions of the lead white crystallites, Mie scattering is expected to be the dominant scattering process^26,27^. Mie scattering is more-or-less wavelength-independent, so it does not affect the color of the pigment. Still, polydisperse lead whites with a submicrometric average particle size (such as the one used in layer β of the *St. Anne* sample), contain a certain fraction of crystallites that have dimensions significantly smaller than the wavelength of visible light (< 100 nm). These crystallites will induce Rayleigh scattering: a process which depends on the wavelength as λ^−4^, causing blue light to be scattered much more efficiently than red light. A demonstration of how a grade of lead white with a submicrometric average crystallite size can exhibit significant Rayleigh scattering is shown in Fig. 6. HC mixed with linseed oil was spread on calibrated test paper with controlled thickness. Two different subtypes of lead white were selected: the first composed of large size crystallites (> 20 µm), the other of submicrometric crystallites (< 400 nm). It was observed that the pigment composed of small particles possessed a strong blue hue, observable in the reflectance spectra (Fig. 6). This result clearly shows an evident optical effect of crystallite size for HC. Still, as the average lead white crystallites—even those in the finer grades—are not expected to exhibit significant Rayleigh scattering, polydispersity will strongly affect the light scattering properties of a batch of lead white. Therefore, two scenarios are proposed, based on the extent to which the lead white in layer β is polydisperse and exhibits Rayleigh scattering.

The first scenario is related to the common observation that typical lead whites have a slightly off-white appearance with a relatively low reflectance towards the shorter wavelengths^28^. Slightly increasing the Rayleigh scattering properties of a batch of lead white could have the effect to increase the observed whiteness of the pigment, which may be desirable for obtaining certain pictorial results. In case of a more significant Rayleigh scattering-related effect, the lead white used in layer β may in fact have exhibited a distinct blue hue, similar to what is shown in Fig. 6. For this alternative scenario, it is relevant to consider the location from which the analyzed sample was taken. It can be seen in Fig. 1 that the sample comes from the foothills of a mountain range of which especially the most distant sections appear slightly blue. In fact, the hue of these distant mountains is very similar to the blue hue in which Da Vinci painted the sky.

In the MS. Leic.—a manuscript written c. 1506–1510—Da Vinci expresses (folio 4r) a very early understanding of how this blue color of the sky is related to the presence of particulate matter in the atmosphere: “*I say that the blueness we see in the atmosphere is not intrinsic color, but is caused by warm vapor evaporated in minute and insensible atoms on which the solar rays fall, rendering them luminous […]*”. He then continues by proposing a simple experiment to mimic this effect with lead white: “*let him paint a board with various colors, among them an intense black […] if he puts on it a very fine and transparent layer of ceruse, he will see that the white of this ceruse will actually be a beautiful blue, but it has to be very fine and well ground*.” It is therefore possible that Da Vinci used a post-processing method in order to give the distant objects in his painting a blue hue according to exactly the same physical phenomenon that would make them appear blue in the real world. A connection can thus be proposed between the observation Leonardo made of the natural surroundings, his theory on color diffusion in the atmosphere, and the processing and selection of his artistic materials.

To see that the idea of painters using Rayleigh scattering to obtain certain pictorial results is not as far-fetched as it may seem, it is interesting to note that the use of a lead white with blue hue scattering properties was previously also hypothesized in Dutch Golden Age paintings^29^—although no experimental results were presented. Moreover, a recent macro X-ray diffraction (MA-XRD) analysis permitted to identify a cerussite-rich subtype of lead white in a specific area of Vermeer’s Girl with a Pearl Earring^30^ which was also hypothesized to have been used in order to obtain specific optical effects. Possibly painters could have observed that washing their pigment in vinegar, in addition to cleaning it from impurities, also gave it a cold blue hue, particularly suited for subtle optical effects. A detailed investigation of the artistic materials needed to obtain such blue hues through Rayleigh scattering was reported by Checroun et al.^31^.

Based on the results presented in this paper, an outlook is here proposed that includes four distinct prospectives. First, it would be of interest to study complementary samples from Leonardo’s paintings to assert whether this specific subtype of lead white was used in comparable paint areas where a blue hue was also desirable. Second, a thorough fundamental PL study of the various inorganic/organic components of ancient paint layers would provide much-needed insight on the interpretation of the data gathered on complex historical samples. While this was recently initiated for the lead white pigments, the PL properties of many other pigments are still not investigated. Third, in terms of instrumental development, it is important to mention that, due to increased commercialization of deep-UV emitting sources, the development of lab-based micro-imaging systems operating below 250 nm are now foreseeable. Also, PL microscopy can be coupled to Raman microscopy^32^, and the chemical selectivity of PL imaging can be increased by time resolution^33,34^. Such developments could dramatically increase the number of applications of PL analysis using lab-based microscopes for the investigation of artistic materials.

Finally, the main limitation of works such as this one, conducted on micro-samples, is the limited representativity of the results. In the case of Leonardo da Vinci, it is actually even more difficult to obtain samples, as the exceptional status of the artworks naturally limits the availability of paint fragments*.* While the use of micro-beams allows to access the complex paint layer build-ups at the micro-scale, by definition the volume of probed mater is very limited. Current developments at C2RMF of non-invasive laboratory instruments, such as a coupled macro-PL and macro-XRF scanner, permitting to collect PL spectra in each pixel of an entire painting surface are thus promising in order to bridge the gap between micro and macro-scale.

## Conclusion

This work explores the different grades of lead white that were used by Leonardo da Vinci to construct his final masterpiece: *The Virgin and Child with St. Anne*. Based on the lead white composition and average crystallite dimensions that were retrieved through Rietveld refinement of HR-XRD data, it is proposed that a post-synthesis treatment was employed on Leonardo’s raw pigments so as to obtain specific pictorial results. These chemical results were found to be in agreement with historical manuscripts written by Leonardo himself, in which he left clues with regards to the use of different grades of lead white. Moreover, complementary SR-µ-PL data obtained on the full stratigraphy reveals how spectral unfolding of hyperspectral PL emission maps can be used to perform semi-quantitative lead white characterization in a spatially resolved manner, without exposing the precious sample to high doses of ionizing radiation.

## Methods

### Synchrotron high-angle resolution X-ray diffraction (SR-HR-XRD)

The two paint fragments α and β were placed in a capillary and rotated in front of a 1 × 1 mm^2^ beam at 35 keV. 20 min scans were repeated up to a total acquisition time of 1–4 h to record XRD patterns with a satisfactory signal to noise ratio. The exploitable angular range was 2 ≤ 2*θ* (°) ≤ 22 (corresponding to 0.93 ≤ d (Å) ≤ 10.14) for the α fragment, allowing the measurement of 152 reflections for C and 49 for HC. The main advantages of this configuration are the excellent angular resolution (0.002°) and the absence of preferential orientation, allowing the collection of high-quality datasets. The exploitable domain was narrower for the β fragment (4 ≤ *2θ* (°) ≤ 14) due to a lower signal-to-noise ratio.

Rietveld analyses of the XRD patterns were carried out with the FullProf Suite^35^, implementing the Thompson-Cox-Hastings profile function with spherical harmonics expansion for the Lorentzian component and axial broadening for the Gaussian in order to model the average microstructure. This allowed to derive a precise composition (error bars of about ± 1 w% estimated from a C/HC calibration mixture) as well as modelling of the morphology and dimensions of the pigment crystallites.

### Synchrotron deep UV-excited photoluminescence (SR-µ-PL)

The embedded *St. Anne* sample was analyzed at the POLYPHEME end-station of the DISCO beamline (Synchrotron SOLEIL, Gif-sur-Yvette, France). The POLYPHEME end-station performs hyperspectral photoluminescence micro-imaging, yielding full emission spectra with high spectral resolution^36,37^. Spectra were recorded with a 40 × /0.6 NA Zeiss Ultrafluar objective, a 250 nm excitation wavelength, an emission wavelength range of 400–680 nm (1.82–3.2 eV), and an integration time of 8 s per spectrum. A map of 30 × 20 pixels was recorded with a step size of 3 µm, covering a total sample area of 90 × 60 µm.

As a first step in the unfolding approach, the positions of the main constituent emission bands were deduced from analysis of the average emission spectrum. In this process, the positions of local minima in the second derivative are taken as positions for the constituent emission bands. For a model making use of *n* Gaussians, the average spectrum is smoothed by convolution with a Gaussian of a width such that the second derivative of the smoothed spectrum exhibits exactly *n* local minima. The width of the Gaussian used for smoothing is found using an automated, iterative process in which each next guess is based on interpolation of all previous guesses. A model consisting of *n* Gaussians is then fitted to the smoothed averaged spectrum to find the width σ of each constituent emission band. The fitting algorithm is based on Newton’s method in optimization, where the residual sum of squares (RSS) is taken as the cost function. Soft parameter limits are implemented by adding a metric to the cost function that rises exponentially beyond, but is close to zero within the limits. Having previously found the positions of the main constituent emission bands and the baseline allows reducing the number of optimizable variables to 2*n* (amplitude and σ). Reducing the number of data points in the spectrum from 512 to 8*n* then allows rapid optimization, even for larger values of *n*, allowing the algorithm to converge several times in a short period of time. Requesting twenty convergences on the same set of variables from different initial guesses then reduces the chance of finding a local, rather than an absolute minimum. The optimization problem can be formally denoted as:

$$\underset{\forall {\varvec{P}}\in {\mathbb{R}}}{\mathrm{min}}\left({\sum }_{i=1}^{8n}{\left({y}_{i}-b-{\sum }_{j=1}^{n}{A}_{j}{e}^{\frac{-{\left({x}_{i}-{\mu }_{j}\right)}^{2}}{2{\sigma }_{j}^{2}}}\right)}^{2}+\frac{1}{10}{\sum }_{k=1}^{2n}\left({e}^{\frac{\left({P}_{k}-{P}_{k}^{min}\right)\mathrm{ln}10}{{P}_{k}^{min}S}}+{e}^{\frac{\left({P}_{k}-{P}_{k}^{max}\right)\mathrm{ln}10}{{P}_{k}^{max}S}}\right)\right)$$

where **P** = **A** ∪ **σ**, forming the set of 2*n* optimizable Gaussian parameters, *b* is the baseline, and *S* is a measure of the softness of the optimization limits. A graphical representation of the multi-step unfolding process is included in the Supplementary Information.

Knowledge of the baseline, the positions and the widths of the main constituent emission bands now allows rapid fitting of the 600 photoluminescence spectra that make up the hyperspectral map. Rather than twenty, three convergences on the same set of parameters are requested per spectrum. The result of this fitting procedure is a set of *n* amplitudes per spectrum, which can be displayed as emission amplitude matrices, resembling the distribution of the compound emitting that particular emission band. It was found that there is high covariance between the different emission amplitude matrices, implying that local absorption and/or reabsorption strongly impacts the emission spectra. In a first approximation, these effects are corrected by normalizing each set of *n* amplitudes *A* to their sum:

$$ {A^{\prime}}_{i}=\frac{{A}_{i}}{{\sum }_{j=1}^{n}{A}_{j}} $$

## Supplementary Information

Supplementary Information

## Data Availability

The datasets generated during and/or analysed during the current study are available from the corresponding author on reasonnable request.
