# Supplementary material for: Microchemical analysis of Leonardo da Vinci’s lead white paints reveals knowledge and control over pigment scattering properties
Source: Sci Rep. 2020 Dec 10;10:21715. doi: 10.1038/s41598-020-78623-5 (PMC7730476; doi:10.1038/s41598-020-78623-5)
Supplement: Supplementary file 1 — Supplementary Information [file 41598_2020_78623_MOESM1_ESM.docx]

Supplementary Information for Microchemical analysis of Leonardo da Vinci’s lead white paints reveals knowledge and control over pigment scattering properties

Victor Gonzalez^*,a,b,c,d^, Selwin Hageraats^*,c,d^, Gilles Wallez^a,b,f^, Myriam Eveno^b,a^, Elisabeth Ravaud^b,a^, Matthieu Refregiers^e^, Mathieu Thoury^d^, Michel Menu^b,a^ and Didier Gourier^a,b^

^a^CNRS, Institut de Recherche Chimie Paris (IRCP), Chimie ParisTech, PSL University, 75005 Paris, France.

^b^Centre de Recherche et de Restauration des Musées de France (C2RMF), Palais du Louvre, 75001 Paris, France.

^c^Rijksmuseum, Science Department, Hobbemastraat 22, 1071 ZC Amsterdam, The Netherlands.

^d^IPANEMA, CNRS, Ministère de la Culture, Université de Versailles Saint-Quentin-en-Yvelines, Université Paris-Saclay, BP48 St. Aubin 91192 Gif-sur-Yvette, France.

^e^Synchrotron SOLEIL, L’Orme des Merisiers, BP48 St. Aubin 91192 Gif-sur-Yvette, France.

^f^Sorbonne Université, UFR926, 75005 Paris, France.

Corresponding author: Victor Gonzalez

**Email:**  [gonzalvic@gmail.com](mailto:gonzalvic@gmail.com)

**1) Rietveld plots** for the samples of the α (upper) and β (lower) layers.


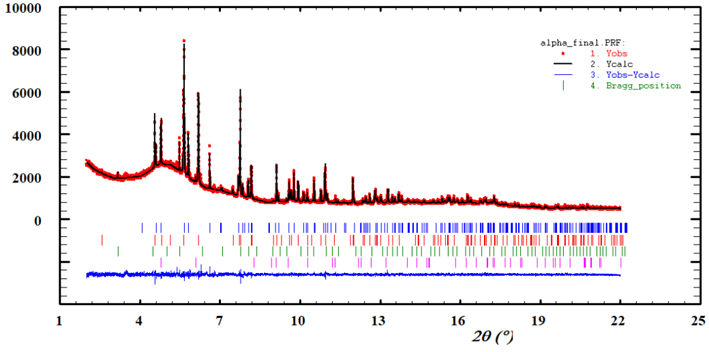


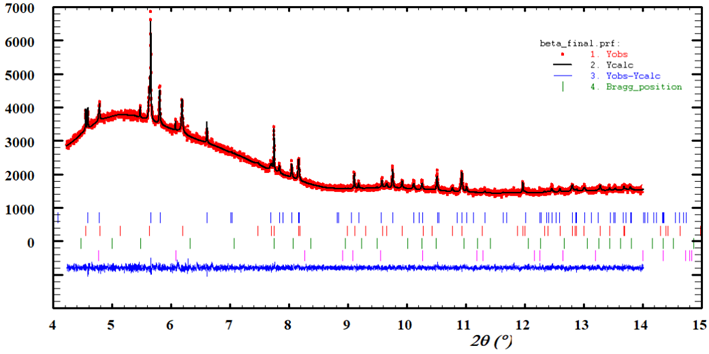


**Figure S1.** Rietveld plots with uncorrected background for the samples of the α (upper) and β (lower) layers. Bars correspond to the identified phases, in the order: cerussite, hydrocerussite, lazurite and quartz.

**2) Rietveld-based quantitative phases analyses**

**sample alpha sample beta**

cerussite 26.68 (0.34) 43.14 (0.93)

hydrocerussite 50.37 (0.58) 36.39 (1.04)

lazurite 21.65 (0.83) 16.03 (1.57)

quartz 1.30 (0.17) 4.44 (0.70)

(value with as-refined su)

**Table S1.** Rietveld-based quantitative phases analyses for samples alpha and beta.

**3) Graphical representation of the multi-step unfolding process**


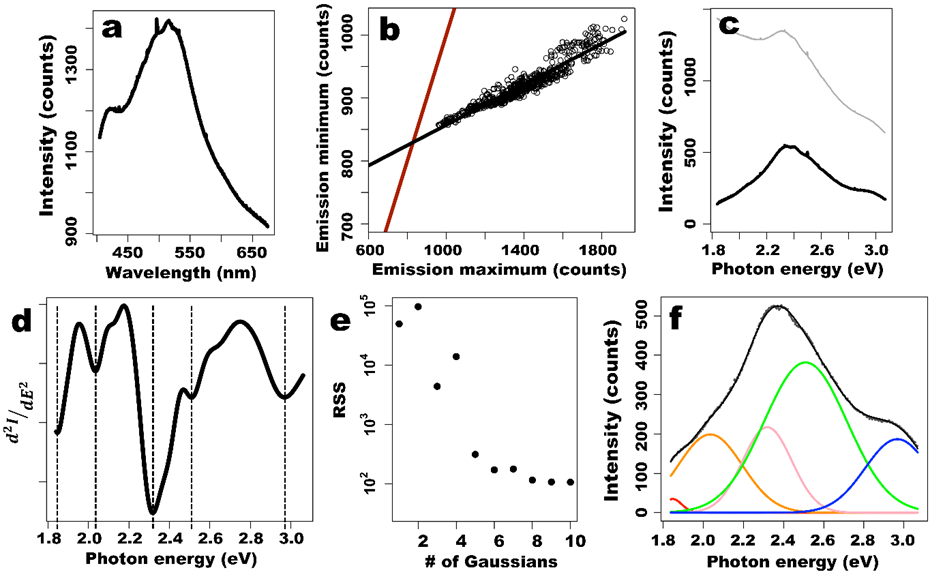


**Figure S2.** a) Average photoluminescence spectrum of the *Ste. Anne* microsample. b) Plot showing the emission minimum of each spectrum as a function of the corresponding emission maximum. The black line shows a linear fit, while the red line shows the points where emission minimum and emission maximum are equal. c) Average emission spectrum after baseline subtraction and Jacobian transformation (black line) and the average emission spectrum after Jacobian transformation, but without baseline subtraction (grey line). d) Second derivative of the average corrected emission spectrum. The black dashed lines indicate minima corresponding to emission band centers. e) RSS as a function of the number of Gaussians used in the model. f) Fit of five Gaussians to the average photoluminescence emission spectrum.

**4) Amplitude matrices for each of the six Gaussian profiles retrieved through the tailored fitting algorithm**.


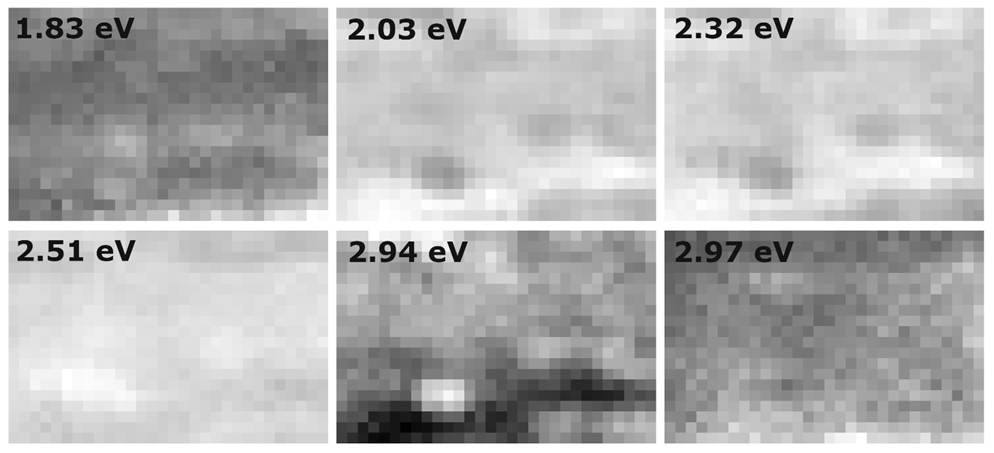


**Figure S3.** Amplitude matrices for each of the six Gaussian profiles retrieved through the tailored fitting algorithm. The energies listed in the upper left corners correspond to the central energy of each Gaussian profile

**5)** **SEM image of the St. Anne sample studied at the DISCO beamline (SOLEIL Synchrotron)**


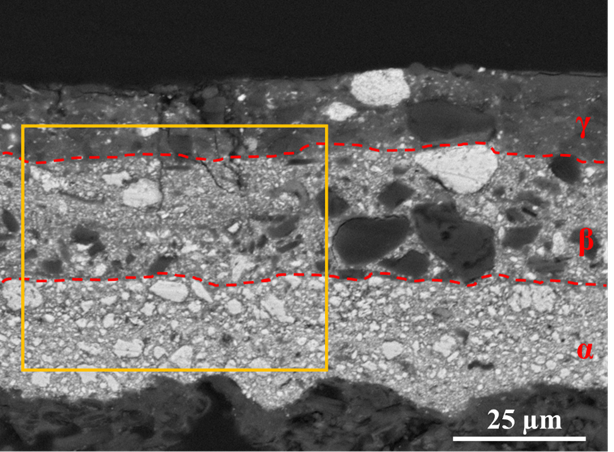


**Figure S4.** SEM image of the St. Anne sample studied at the DISCO beamline. The gesso layer is visible on this image, below layer α. The orange rectangle corresponds to the area analyzed via SR-µ-PL.

**6)** **Recapitulative of the structural and optical data for each layer**

|  | **Layer alpha** | | | **Layer beta** | | |
| --- | --- | --- | --- | --- | --- | --- |
|  | **C** | **HC** | **R=(HC/HC+C)** | **C** | **HC** | **R** |
| **SR-HR-XRD** | 26.68±0.34 | 50.37±0.58 | 65±2 w% | 43.14±0.93 | 36.39±1.04 | 46±2 w% |
|  | | | | | | |
| **SR-µ-PL** | **A_C_** = 42.3±1.1 | **A_HC_** = 173.5±0.4 |  | **A_C_ =** 127.3±1.4 | **A_HC_** = 245.9±0.3 |  |

**Table S2.** XRD measured HC:C ratios and PL amplitudes for each layer
